# The oral - X axis: from periodontal dysbiosis to systemic disease

**DOI:** 10.3389/fimmu.2026.1806445

**Published:** 2026-04-29

**Authors:** Wenqin Jin, Lichao Tang, Jiaqi Yang, Xianlong Hu, Weiwei Guo, Huangping Ai, Yuling Zuo, Zhao Jin

**Affiliations:** 1 Chengdu University of Traditional Chinese Medicine, Chengdu, Sichuan, China; 2 Hospital of Chengdu University of Traditional Chinese Medicine, Chengdu, Sichuan, China

**Keywords:** immune dysregulation, inflammatory overflow, oral - X axis, oral pathogens, oxidative stress

## Abstract

Chronic oral inflammatory diseases, particularly periodontitis, are increasingly recognized as important contributors to the onset and progression of systemic disorders. Accumulating epidemiological, clinical, and mechanistic evidence indicates that the oral cavity is not an isolated organ, but rather a critical hub and early window for systemic disease development. Through microbial translocation, chronic low-grade inflammation, immune dysregulation, oxidative stress, and epigenetic reprogramming, oral diseases engage in bidirectional communication with distant organs.We conceptualize this integrated network as the “oral-X axis, “ encompassing the oral-cardiovascular, oral-metabolic, oral–respiratory, oral–gastrointestinal, oral-oncologic, oral-immune, oral–brain, and other systemic axes. At the core of these interactions lies periodontitis-associated microbial dysbiosis dominated by key pathogens such as Porphyromonas gingivalis, Fusobacterium nucleatum, and Aggregatibacter actinomycetemcomitans. The ensuing inflammatory response compromises periodontal barrier integrity, facilitating the dissemination of bacteria, virulence factors, and inflammatory mediators into the systemic circulation. These processes promote endothelial dysfunction, insulin resistance, breakdown of immune tolerance, neuroinflammation, and the formation of pro-tumorigenic microenvironments, thereby mechanistically linking oral inflammation to a broad spectrum of systemic diseases. This review systematically summarizes the current evidence supporting the oral-X axis, with a particular focus on epidemiological associations and underlying molecular and cellular mechanisms. In addition, we discuss periodontal interventions and oral microbiome modulation as potential strategies for the prevention and treatment of systemic diseases. A deeper understanding of the oral-X axis may provide novel insights into integrated oral–systemic healthcare and precision medicine.

## 1 Introduction

Chronic periodontitis (CP) is a chronic inflammatory disease driven by the interaction between dental plaque-associated microorganisms and host immune responses. Sustained inflammation and immune activation lead to the progressive destruction of periodontal supporting tissues and alveolar bone resorption (1). Epidemiological studies indicate that periodontitis is the sixth most prevalent disease worldwide, with its global burden continuing to increase due to population aging and the persistence of major risk factors. It is estimated that more than 10% of adults globally are affected by severe periodontal disease (PD) (2).

An expanding body of evidence has demonstrated strong associations between oral diseases and a wide range of systemic disorders, including diabetes mellitus, inflammatory bowel disease, colorectal cancer, Parkinson’s disease, Alzheimer’s disease, atherosclerosis, adverse pregnancy outcomes, thyroid cancer, rheumatoid arthritis, and systemic lupus erythematosus (3–9). Shared pathogenic pathways underlying the interplay between oral and systemic diseases include chronic low-grade inflammation, microbial dysbiosis, immune dysregulation, oxidative stress, and metabolic imbalance. In addition, periodontal inflammation facilitates the systemic dissemination of oral pathogens, virulence factors, and inflammatory mediators. Conversely, systemic diseases may exacerbate periodontal tissue destruction by disrupting immune and metabolic homeostasis, highlighting the bidirectional relationship between oral health and systemic health.

Accordingly, we propose the concept of the “oral-X axis” to describe the bidirectional biological communication between the oral cavity and multiple organ systems. Within this framework, the oral cavity functions as a major microbial reservoir and inflammatory source, linking oral health to systemic pathophysiology through multiple dissemination routes and signaling pathways. This review aims to systematically summarize current research on the oral-X axis, integrating epidemiological evidence with underlying molecular and cellular mechanisms, and to discuss the clinical implications of oral interventions and modulation strategies in the prevention and management of systemic diseases.

## 2 Current research situation of “oral-X” axis

### 2.1 Oral-cardiovascular axis

Cardiovascular disease (CVD) remains the leading cause of non-communicable mortality worldwide, accounting for approximately one-third of all global deaths (10). Epidemiological studies indicate that the prevalence of both hypertension and periodontitis increases in parallel with advancing age, and that the two conditions share common demographic and behavioral risk factors, including older age, male sex, smoking, overweight status, and metabolic disturbances (3, 11–13). Genetic studies further suggest that interactions between polymorphisms in endothelin-1 (ET-1), angiotensin-converting enzyme (ACE), and pro-inflammatory cytokines (e.g., TNF-β) may increase susceptibility to periodontitis (14). Conversely, individuals with moderate to severe periodontitis exhibit a 20-50% higher risk of hypertension compared with periodontally healthy individuals (15).

Atherosclerosis (AS), the pathological basis of most forms of CVD, shares substantial overlap with periodontitis in terms of inflammatory and oxidative mechanisms, including elevated C-reactive protein levels, cytokine imbalance, and enhanced oxidative stress (16–21). Epidemiological evidence demonstrates that periodontitis is associated with an approximately 20% increased risk of atherosclerosis, while longitudinal studies further suggest that periodontal disease may elevate the future risk of atherosclerotic cardiovascular events (22–24).

### 2.2 Oral-respiratory axis

An increasing body of evidence supports a close association between periodontitis and respiratory diseases, particularly pneumonia. Patients with pulmonary disorders often exhibit poorer periodontal status, characterized by higher periodontal indices and an increased risk of oral infection (25). Clinical studies have demonstrated that individuals with moderate to severe periodontitis have an approximately fourfold higher risk of developing community-acquired pneumonia (CAP) compared with periodontally healthy individuals (26).

Notably, a two-sample Mendelian randomization study conducted in East Asian populations provided evidence for a potential causal relationship between specific oral microbial genera and respiratory tract infections, further revealing species-specific effects on bronchitis, pneumonia, sinusitis, and bronchiectasis (27). Long-term observational studies have also shown that periodontitis is associated with increased pneumonia-related mortality in vulnerable populations, such as patients undergoing hemodialysis (26, 28, 29).

### 2.3 Oral-metabolic axis

Diabetes mellitus is a global metabolic disorder affecting more than 440 million individuals worldwide and is characterized by chronic hyperglycemia and systemic inflammation (30). Diabetes markedly increases the risk of periodontitis, with the prevalence of periodontal disease in diabetic patients being approximately threefold higher than in non-diabetic individuals (31, 32). Conversely, periodontitis exacerbates glycemic dysregulation, establishing a bidirectional pathogenic relationship. Clinical and experimental evidence indicates that periodontal therapy can significantly reduce glycated hemoglobin levels, systemic inflammatory burden, and vascular dysfunction, with effects comparable to those achieved by the addition of an antidiabetic medication (33–35). A recent cross-sectional clinical study further demonstrated that, among patients who were unaware of their diabetic status, chairside HbA1c testing during periodontal treatment can detect previously unrecognized hyperglycemia. This finding indicates that periodontitis is not only closely associated with metabolic diseases but can also serve to monitor early disturbances in glucose metabolism (36).

Pregnancy-related metabolic disturbances are also closely associated with periodontal disease. Severe periodontitis has been independently linked to an increased risk of gestational diabetes mellitus (GDM) (37–42). Obesity further amplifies periodontal inflammation during pregnancy, suggesting that preconception weight management and periodontal intervention may help attenuate systemic inflammatory activation (43).

In addition, polycystic ovary syndrome (PCOS), a heterogeneous endocrine–metabolic disorder, has been consistently associated with periodontal inflammation and oxidative stress (44–51). Meta-analyses have demonstrated a bidirectional relationship, whereby periodontitis increases the risk of PCOS by 46%, and PCOS increases the risk of periodontitis by 28% (51). Hypothyroidism and obesity have also been reported to be associated with periodontitis, sharing common features of chronic low-grade inflammation and increased periodontal susceptibility (52–62).

### 2.4 Oral-gastrointestinal axis

Inflammatory bowel disease (IBD), including Crohn’s disease and ulcerative colitis, is frequently accompanied by oral manifestations and an increased prevalence of periodontitis (63–79). Meta-analyses have demonstrated that both the prevalence and severity of periodontitis are significantly higher in patients with IBD than in healthy controls, suggesting the involvement of shared immune-mediated mechanisms between these conditions (79–82).

Chronic liver diseases (CLD), encompassing viral hepatitis, non-alcoholic fatty liver disease (NAFLD), and liver cirrhosis, have also been associated with periodontal disease (83–86). Patients with NAFLD exhibit a higher prevalence of periodontitis, and interventional studies indicate that periodontal therapy can reduce serum liver enzyme levels as well as systemic antibody titers against periodontal pathogens (87–89). Moreover, liver cirrhosis and post–liver transplantation status are associated with exacerbated periodontal inflammation and an increased incidence of peri-implant complications, underscoring the bidirectional interplay between oral and hepatic health (90–94).

### 2.5 Oral-oncologic axis

Periodontitis is increasingly recognized as a potential risk factor for multiple malignancies, particularly cancers of the digestive tract. Oral squamous cell carcinoma has been shown to be associated with periodontal inflammation and dysbiosis, with periodontal pathogens frequently enriched within tumor tissues (95–99). Epidemiological studies further support associations between periodontitis and cancers of the esophagus, stomach, colorectum, liver (hepatocellular carcinoma), and pancreas, which remain statistically significant after adjustment for major confounding factors such as smoking, alcohol consumption, and diabetes (100–114).

In addition, associations between periodontitis and lung, thyroid, and breast cancers have been reported; however, the strength and consistency of evidence vary substantially across different cancer types (115–124). Overall, chronic periodontal inflammation and microbial dysbiosis may contribute to tumor initiation and progression through systemic inflammatory and immune-mediated pathways, although causal relationships have yet to be definitively established.

### 2.6 Oral-immune axis

Periodontitis shares fundamental mechanisms of chronic inflammation and immune dysregulation with immune-mediated diseases. Among these, the association between rheumatoid arthritis (RA) and periodontitis is particularly prominent, with up to 75% of patients with RA exhibiting moderate to severe periodontal disease (125–129). Meta-analyses indicate that periodontitis increases the risk of RA by approximately 69%, and that RA disease severity is positively correlated with the extent of periodontal destruction.

Similarly, systemic lupus erythematosus (SLE) and Graves’ disease have been associated with periodontal inflammation, alterations in the oral microbiota, and elevated levels of pro-inflammatory cytokines, further supporting a contributory role of periodontal disease in systemic immune activation (130–135).

### 2.7 Oral-brain axis

Neurodegenerative and neuropsychiatric disorders have emerged as a critical component of the oral–X axis. Chronic periodontitis has been associated with an increased risk of Alzheimer’s disease, with elevated antibodies against periodontal pathogens detectable several years prior to cognitive decline (136–139). Parkinson’s disease has also been linked to periodontal inflammation, and longitudinal studies suggest that sustained periodontal care may slow disease progression (140–143). Furthermore, periodontitis has been identified as an independent risk factor for depression, potentially mediated through systemic inflammation and behavioral pathways (144–148).

### 2.8 Oral-other axis

Periodontal inflammation in pregnant women has been associated with adverse pregnancy outcomes (149), with approximately 40% of expectant mothers exhibiting clinical signs of periodontitis (150). Evidence indicates that periodontitis may increase the risk of preterm birth (151), spontaneous abortion (152), low birth weight (153), preeclampsia (154), fetal growth restriction (155), and gestational diabetes mellitus (156).

Chronic kidney disease (CKD) is defined as structural or functional abnormalities of the kidney persisting for more than three months (157). Numerous population-based studies have demonstrated a close association between periodontitis and CKD. In a cross-sectional study of middle-aged adults (n = 5, 537), Kshirsagar et al. reported that individuals with chronic periodontitis exhibited an estimated glomerular filtration rate decline approximately twice that of periodontally healthy individuals, with kidney damage severity positively correlating with periodontitis severity (158). A longitudinal study involving 1, 486 participants confirmed that, after adjusting for age, sex, diabetes, hypertension, and smoking, periodontal pocket depth remained independently associated with CKD progression (159). Another cohort study of 761 men found that the prevalence of CKD in participants with severe periodontitis was 2.04-fold higher than in controls (160). These findings suggest that periodontal inflammation may contribute to renal impairment via persistent systemic inflammation and immune dysregulation.

Prostatitis is clinically characterized by urinary symptoms and genital pain, with a prevalence of up to 25% (161). Epidemiological evidence indicates that periodontitis is associated with an increased risk of benign prostatic hyperplasia (BPH), with odds ratios ranging from 1.50 to 1.68 after adjusting for clinical and demographic confounders (162, 163). Prostate cancer (PC) is the second most common malignancy among men worldwide (164). Cohort studies across different regions have consistently shown that chronic periodontitis modestly but significantly increases PC risk, with relative risk elevations ranging from 14% to 73%, depending on study design and follow-up duration (165–167).

**Figure 1:**
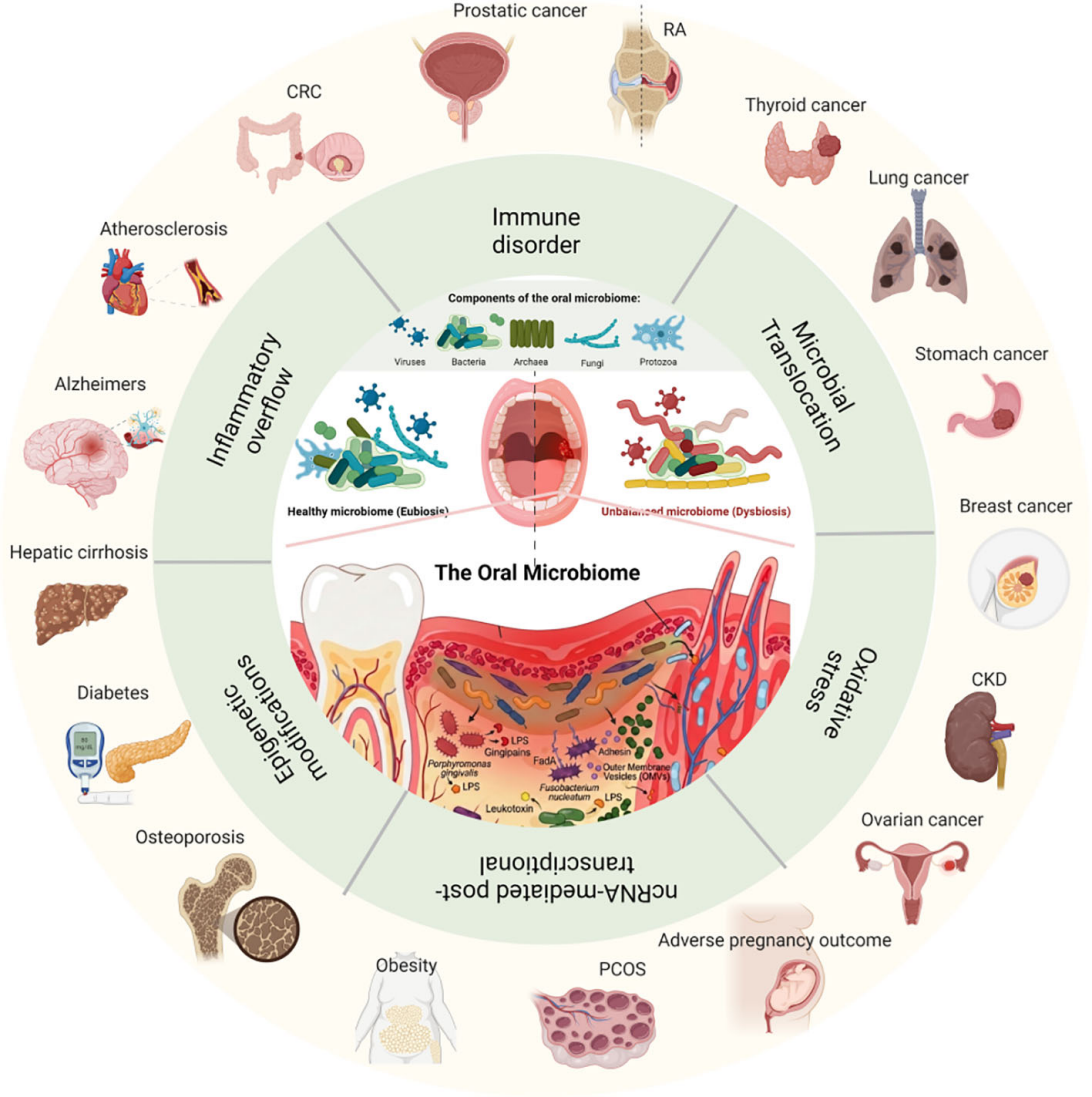
The oral - X axis: from periodontal dysbiosis to systemic disease.

Osteoporosis (OP) is a systemic skeletal disorder characterized by decreased bone mass and increased fracture risk, predominantly affecting postmenopausal women and the elderly (168). Accumulating evidence suggests a bidirectional relationship between periodontitis and bone loss. In a study of 5, 383 participants, Tak et al. reported a significant correlation between the number of remaining teeth in men and bone mineral density (BMD) at the lumbar spine and proximal femur, with individuals possessing fewer than 10 teeth exhibiting markedly lower BMD (169). Other studies have shown that osteoporosis increases the risk of periodontitis by more than twofold (170), while hormone replacement therapy combined with vitamin D and calcium supplementation can mitigate periodontal disease severity in osteoporotic women (171). Conversely, severe periodontitis has been associated with increased osteoporosis incidence (172); a large cohort study reported that periodontitis was an independent risk factor for osteoporosis in women (HR = 1.22, 95% CI: 1.01–1.48), whereas no such association was observed in men (173). These findings support the notion that periodontal disease and skeletal disorders share common inflammatory and bone remodeling pathways (**Table 1**, **Figure 1**).

**Table 1:** Epidemiological evidence and mechanistic overview of the oral–X axis and systemic diseases.

Oral–X axis	Associated systemic diseases	Key epidemiological/clinical evidence	Representative mechanisms
Oral–cardiovascular axis	Hypertension, atherosclerosis, cardiovascular disease (CVD)	Periodontitis is associated with a 20–50% increased risk of hypertension and approximately a 20% higher risk of atherosclerosis; both conditions share common demographic characteristics and genetic susceptibility	Systemic inflammation, endothelial dysfunction, oxidative stress, imbalance of inflammatory mediators
Oral–respiratory axis	Pneumonia, Bronchitis, Sinusitis, Bronchiectasis	Moderate-to-severe periodontitis is associated with an approximately fourfold increased risk of community-acquired pneumonia; Mendelian randomization studies support a causal role of oral microorganisms	Aspiration of oral pathogens, airway colonization, impaired host defense
Oral–metabolic axis	Diabetes, Gestational diabetes mellitus (GDM), Obesity, Polycystic Ovary Syndrome (PCOS), Thyroid diseases	The prevalence of periodontitis is approximately threefold higher in patients with diabetes; periodontal therapy significantly reduces HbA1c levels, with an effect size comparable to adjunctive antidiabetic medication; a bidirectional association with PCOS has been reported	Low-grade systemic inflammation, insulin resistance, oxidative stress, endocrine–immune interactions
Oral–gut axis	Inflammatory bowel disease (IBD), Non-alcoholic fatty liver disease (NAFLD), Chronic liver disease, Liver cirrhosis	Patients with IBD exhibit increased prevalence and severity of periodontitis; periodontal treatment has been shown to improve liver enzyme levels and immunological parameters in patients with NAFLD	Mucosal immune dysregulation, microbial translocation, oral–gut microbiota crosstalk
Oral–cancer axis	Oral cancer, Esophageal cancer, Gastric cancer, Colorectal cancer, Pancreatic cancer, Liver cancer	After adjustment for smoking and alcohol consumption, periodontitis remains significantly associated with an increased risk of gastrointestinal malignancies; periodontal pathogens are enriched in tumor tissues	Chronic inflammation, immune modulation, microbe-mediated carcinogenesis
Oral–immune axis	Rheumatoid arthritis (RA), Systemic lupus erythematosus (SLE), Graves’ disease	Periodontitis is associated with an approximately 69% increased risk of RA; disease activity correlates with the severity of periodontal tissue destruction	Molecular mimicry, autoantibody production, trained immunity
Oral–brain axis	Alzheimer’s disease, Parkinson’s disease, Depression	Elevated antibody levels against periodontal pathogens precede cognitive decline; periodontal intervention may slow the progression of Parkinson’s disease	Neuroinflammation, systemic cytokine signaling, blood–brain barrier dysfunction
Oral–pregnancy axis	Adverse pregnancy outcomes	Periodontitis is associated with increased risks of preterm birth, miscarriage, low birth weight, preeclampsia, and GDM	Inflammatory overflow, placental immune activation
Oral–kidney axis	Chronic kidney disease (CKD)	Periodontitis is associated with an approximately twofold increased risk of estimated glomerular filtration rate (eGFR) decline and accelerated CKD progression	Persistent systemic inflammation, immune dysregulation
Oral–prostate axis	Benign prostatic hyperplasia (BPH), Prostate cancer (PC)	Periodontitis is associated with an increased risk of BPH (OR 1.50–1.68) and a modestly elevated risk of prostate cancer	Chronic inflammation, immune modulation
Oral–bone metabolism axis	Osteoporosis	A bidirectional association between periodontitis and reduced bone mass has been reported, with notable sex-specific differences	Inflammatory bone remodeling, dysregulated osteoimmune signaling

CVD, cardiovascular disease; CAP, community-acquired pneumonia; GDM, gestational diabetes mellitus; PCOS, polycystic ovary syndrome; IBD, inflammatory bowel disease; NAFLD, non-alcoholic fatty liver disease; RA, rheumatoid arthritis; SLE, systemic lupus erythematosus; CKD, chronic kidney disease; eGFR, estimated glomerular filtration rate; BPH, benign prostatic hyperplasia; PC, prostate cancer; OR, odds ratio.

## 3 Pathophysiological mechanisms of the oral-X axis

### 3.1 Oral microbial dysbiosis as an initiating factor

The oral cavity is one of the four major microbial reservoirs in the human body, and disruption of its homeostasis is central to the development and progression of periodontitis. Key periodontal pathogens, including Porphyromonas gingivalis, Fusobacterium nucleatum, and Aggregatibacter actinomycetemcomitans, produce virulence factors such as lipopolysaccharides (LPS), gingipains, leukotoxins, outer membrane vesicles (OMVs), and adhesins (e.g., FadA), which continuously compromise the local epithelial barrier and induce inflammation. This facilitates systemic dissemination of microbial components, contributing to multisystem diseases. For example, the Cnm^+^ serotype of Streptococcus mutans, which exhibits enhanced extracellular matrix (ECM) binding, has been associated with infective endocarditis, cerebrovascular disease, and IgA nephropathy (174–182). Fusobacterium nucleatum is enriched in oral squamous cell carcinoma tissues and increases with tumor progression (183, 184). Subgingival colonization by Tannerella forsythia, Campylobacter rectus, Prevotella intermedia, Prevotella melaninogenica, and P. gingivalis during pregnancy has been linked to preterm birth and low birth weight (185–188). The abundance of A. actinomycetemcomitans, P. gingivalis, T. forsythia, and Treponema denticola is significantly associated with hypertension, and P. gingivalis antigens can exacerbate angiotensin II–induced hypertensive responses (189–192). Increased oral pathogen load and reduced microbial diversity in patients with systemic lupus erythematosus, chronic kidney disease, and esophageal cancer further indicate the systemic impact of oral dysbiosis (132, 193–196).

### 3.2 Ectopic colonization of oral pathogens

Ectopic colonization of oral pathogens refers to the dissemination of oral microorganisms to distant organs via swallowing, inhalation, transient bacteremia, or immune cell–mediated transport, and constitutes an important pathological basis of the oral–systemic disease axis (197–199).

#### 3.2.1 Gastrointestinal route

Approximately 1.5 L of saliva enters the digestive tract daily. When gastrointestinal barrier integrity is compromised, P. gingivalis, F. nucleatum, Klebsiella spp., and other pathogens can survive and colonize the gut, disrupting microbial homeostasis, reducing short-chain fatty acid production, impairing epithelial barriers, and increasing the risk of inflammatory bowel disease (IBD) and colorectal cancer (200–202). A. actinomycetemcomitans and P. gingivalis have been associated with chronic atrophic gastritis and gastric precancerous lesions (203). In animal models, P. gingivalis can migrate via the pancreatic duct and promote pancreatic tumorigenesis (204). Oral-origin F. nucleatum is enriched in colorectal cancer tissues, driving pro-tumor inflammation and remodeling the immune microenvironment (205–216).

#### 3.2.2 Respiratory route

Periodontal pathogens can be aspirated into the lower respiratory tract, inducing pneumonia and exacerbating chronic obstructive pulmonary disease (COPD). Dental plaque and periodontal pockets serve as reservoirs for Streptococcus pneumoniae, Klebsiella pneumoniae, and Pseudomonas aeruginosa (25, 217–222). Typical periodontal bacteria have been detected in airway samples of patients during acute exacerbations of severe COPD, indicating oral–respiratory microbial crosstalk (217).

#### 3.2.3 Hematogenous dissemination

Ulcerated periodontal pockets provide a direct pathway for bacteria and endotoxins to enter the circulation. Recurrent transient bacteremia in animal models accelerates coronary and aortic atherosclerosis, supporting a causal link between periodontal infection and cardiovascular disease (19, 223–225).

#### 3.2.4 Placental and genitourinary route

High-throughput sequencing has shown that placental microbiota more closely resembles the oral microbiome than the gut or vaginal microbiomes. F. nucleatum, P. gingivalis, and C. rectus have been detected in placental tissues and amniotic fluid, inducing inflammatory cascades, impairing placental function, and contributing to adverse pregnancy outcomes (226–238). Oral pathogens may also reach the reproductive tract via the gastrointestinal–reproductive axis or sexual transmission, further disrupting local microbial homeostasis (239, 240).

#### 3.2.5 Neural route

Postmortem brain tissues from Alzheimer’s disease patients have revealed components of P. gingivalis LPS, Treponema spp., and Chlamydia pneumoniae. Animal studies have similarly detected P. gingivalis in mouse brains, suggesting its ability to cross the blood–brain barrier and participate in neurodegenerative processes (241–245).

#### 3.2.6 Urogenital route

Studies have detected periodontal pathogens such as T. denticola, P. gingivalis, and F. nucleatum in subgingival plaque, prostatic secretions, and prostate cancer tissues of patients with chronic periodontitis, chronic prostatitis, and benign prostatic hyperplasia, supporting the involvement of ectopic oral colonization in urogenital pathology (246, 247).

#### 3.2.7 Immune cell-mediated transport

Periodontal pathogens can also persist within macrophages and neutrophils, exploiting an immune cell-mediated “Trojan horse” mechanism to disseminate systemically (248).

### 3.3 Inflammatory overflow

Periodontitis is a chronic inflammatory disease driven by dysbiosis, characterized by sustained immune activation and the release of substantial inflammatory mediators. Patients exhibit persistently elevated levels of circulating CRP, IL-1β, IL-6, and TNF-α, resulting in low-grade systemic inflammation. This state serves as the “common soil” for various systemic chronic diseases (249–251).

#### 3.3.1 Metabolic abnormalities

A significant synergistic amplification effect exists between periodontitis and obesity. In obese patients with chronic periodontitis, the proportion of Tannerella forsythia in the subgingival biofilm increases with rising BMI (252). Furthermore, obese women with periodontitis demonstrate a significantly higher detection rate of Porphyromonas gingivalis in the gingival sulcus compared to non-obese periodontitis patients (253). Additionally, obesity-related metabolic abnormalities, such as insulin resistance, decreased insulin sensitivity, and dyslipidemia are considered critical risk factors for the onset and progression of periodontal disease. The degree of insulin resistance is closely correlated with clinical parameters such as periodontal attachment loss, probing depth, and bleeding index (254). Patients with combined obesity and insulin resistance exhibit significantly greater periodontitis severity than those with obesity alone (255). The underlying mechanism involves inflammation and oxidative stress, which impair insulin sensitivity and lead to the accumulation of glycolysis products in the gingival crevicular fluid (GCF), thereby exacerbating periodontal tissue destruction (255–257). In patients with metabolic syndrome (MetS), gingival crevicular fluid (GCF) biomarkers exhibit distinct patterns. Aggrecan levels decrease with increasing severity of periodontitis, reflecting local tissue degradation. In contrast, IL-8 and MMP-8 levels are elevated in patients with diabetes independently of periodontal status, indicating systemic metabolic inflammation. These patterns support the use of GCF analysis as a non-invasive tool for probing oral-systemic interactions and suggest that, in populations with metabolic dysfunction, periodontal therapy and glycemic control may exert synergistic beneficial effects on the GCF inflammatory profile (258).

#### 3.3.2 Cardiovascular disease

Periodontitis is an independent, non-traditional risk factor for cardiovascular disease (CVD), a prevalent association that is particularly significant in adults under 45 and middle-aged women (259, 260). Low-grade systemic inflammation is considered a crucial mechanistic link between periodontitis and atherosclerosis. Circulating inflammatory mediators and periodontal pathogen endotoxins can activate vascular endothelial cells, inducing endothelial dysfunction, one of the initiating steps of atherosclerosis (261). Within this inflammatory environment, leukocyte adhesion, smooth muscle cell migration, and lipid deposition are accelerated, promoting arterial plaque formation. Concurrently, the upregulation of matrix metalloproteinases (MMPs) weakens the stability of the fibrous cap, increasing the risk of plaque rupture and thrombosis, which may precipitate myocardial infarction or stroke. High-sensitivity CRP (hs-CRP), as a comprehensive marker of systemic inflammatory burden, has been proposed as a vital reference indicator for future periodontal grading and risk assessment (262).

#### 3.3.3 Kidney injury

Patients with chronic kidney disease (CKD) universally exhibit a micro-inflammatory state, characterized by elevated levels of pro-inflammatory markers such as IL-6, CRP, TNF-α, and fibrinogen (263). Studies indicate that serum CRP levels are significantly elevated in periodontitis patients, and circulating periodontal pathogens and their byproducts can directly injure renal endothelial cells. Consequently, periodontitis, as a source of chronic inflammation, may promote the onset and progression of CKD (264). Furthermore, periodontitis is closely associated with endothelial dysfunction. Bacteria and inflammatory mediators disseminate continuously via the bloodstream to distal organs like the kidneys, disrupting microvascular endothelial homeostasis and aggravating renal injury, which further supports the risk role of periodontitis in CKD pathogenesis (265).

#### 3.3.4 Neuroinflammation

Systemic inflammation triggered by periodontitis can activate microglia in the central nervous system via increased blood–brain barrier (BBB) permeability or vagus nerve signaling, thereby inducing neuroinflammatory responses. Activated microglia release neurotoxic mediators, leading to neuronal injury and synaptic dysfunction—processes closely implicated in the pathogenesis of depression and neurodegenerative diseases (266–268). Moreover, periodontitis-associated inflammatory cytokines (e.g., IL-1β, TNF-α) can interfere with the metabolism of neurotransmitters such as serotonin and dopamine, resulting in mood dysregulation and an increased risk of depression (265, 269, 270).

#### 3.3.5 Adverse pregnancy outcomes

Periodontal pathogens can disrupt epithelial and endothelial barriers, inducing local placental inflammation, abnormal remodeling of spiral arteries, and placental functional defects, which may lead to adverse pregnancy outcomes (271–278). Additionally, inflammatory mediators (such as TNF-α, IL-6, and PGE2) can directly stimulate uterine myometrial contraction, promoting the premature rupture of membranes (PROM) and preterm birth; alternatively, they may cross the placental barrier to induce fetal immune-inflammatory responses, causing damage to fetal tissues and organs (279–288).

### 3.4 Oxidative stress

Oxidative stress is recognized as a crucial pathophysiological bridge linking localized periodontal inflammation to systemic diseases. During the onset and progression of periodontitis, neutrophils undergo a “respiratory burst” to eliminate subgingival pathogens, generating a large amount of reactive oxygen species (ROS). When local ROS production exceeds the clearance capacity of the body’s antioxidant system, excess ROS can enter the systemic circulation, triggering a systemic oxidative stress response. This leads to lipid peroxidation, protein modification, and DNA damage, thereby contributing to the pathogenesis of various systemic diseases.

Clinical studies have shown that the level of oxidative stress in patients with type 2 diabetes mellitus (T2DM) and periodontitis is significantly higher than in individuals with periodontitis alone or in healthy individuals, with a marked decrease in plasma small-molecule antioxidant capacity (289). Pradeep et al. (290) reported that levels of 4-hydroxy-2-nonenal (4-HNE), a terminal product of lipid peroxidation, in both gingival crevicular fluid and serum, were significantly higher in patients with T2DM and chronic periodontitis compared to patients with periodontitis alone.

Oxidative stress also serves as a significant shared mechanism between periodontitis and polycystic ovary syndrome (PCOS). Chronic periodontitis can lead to elevated levels of DNA oxidative damage markers in the serum and saliva of PCOS patients, alongside a reduction in total serum antioxidant status (291).

When periodontitis coexists with atherosclerosis, the body’s oxidative load is significantly higher than in either condition alone. Clinical evidence indicates that serum total oxidant status (TOS) and oxidative stress index (OSI) are markedly elevated in periodontitis patients with atherosclerosis, while antioxidant capacity is further compromised (292).

In recent years, mitochondrial dysfunction has been identified as an important cytological basis for oxidative stress in periodontitis. Gingival tissues and gingival fibroblasts from patients with chronic periodontitis commonly exhibit mitochondrial structural abnormalities, decreased membrane potential, reduced oxygen consumption rate, and a decreased copy number of mitochondrial DNA. Stimulation with P. gingivalis LPS can induce healthy gingival fibroblasts to prematurely generate mitochondrial ROS, triggering the release of IL-1β, IL-6, and TNF-α. This amplified inflammatory cascade can be effectively blocked by mitochondria-targeted antioxidants or by enhancing endogenous antioxidant enzyme activity (293, 294).

### 3.5 Immune dysregulation and the pro-tumorigenic microenvironment

Oral chronic inflammation can remodel host immunity, leading to a prolonged imbalance in immune homeostasis. Through pathological immune activation, immune suppression/exhaustion, and immune cell recruitment and polarization, it significantly increases susceptibility to autoimmune diseases, chronic inflammatory diseases, and malignancies.

#### 3.5.1 Autoimmune reactions and immune activation

Multiple studies have indicated that Porphyromonas gingivalis (P. gingivalis) infection often precedes the onset of rheumatoid arthritis (RA), and patients with aggressive periodontitis exhibit significantly elevated anti-citrullinated protein antibody (ACPA) titers (295–297). The autoimmune reaction to citrullinated proteins is a fundamental basis for RA pathogenesis. P. gingivalis is currently the only known oral pathogen capable of producing peptidylarginine deiminase (PAD). It can break self-tolerance via “molecular mimicry, “ inducing citrullination of various host proteins, thereby triggering the generation of ACPAs. These ACPAs cross-react with synovial antigens, driving synovitis and joint destruction in RA (298, 299).

#### 3.5.2 Systemic humoral immune activation

Patients with periodontitis show significantly elevated local and systemic levels of IgA/IgG. Moreover, antibody titers against periodontal pathogens are positively correlated with antibodies against malondialdehyde-acetaldehyde modified LDL, which is closely associated with the severity of atherosclerosis (18, 300–303). Systemic IgG levels are significantly increased in mice infected with Filifactor alocis (304). The abundance of Anaeroglobus geminatus positively correlates with probing depth and the anti-phosphatidylcholine IgG response, suggesting its involvement in driving systemic humoral immune activation during periodontitis (132, 299).

#### 3.5.3 Tumor immune remodeling driven by periodontitis

##### 3.5.3.1 Immune suppression and T cell dysfunction

Periodontal pathogens have been detected in tissues of oral squamous cell carcinoma (OSCC), esophageal cancer, pancreatic cancer, and colorectal cancer (CRC). Long-term chronic antigen stimulation may lead to T cell exhaustion or immune tolerance, weakening the body’s immune surveillance against other pathogens or tumor cells. Clinical studies show that PD-1/PD-L1 expression in the peripheral blood and tumor microenvironment of periodontitis patients is significantly higher than in healthy controls, and this phenomenon can be partially reversed by initial periodontal therapy (305). Fecal microbiota transplantation experiments further confirmed that the periodontitis-associated microbiota can downregulate IFN-γ^+^ CD8^+^ T cells in mice, accelerating CRC development (306). In CRC samples, Fusobacterium nucleatum (F. nucleatum) DNA content is negatively correlated with CD3^+^ T cell density. F. nucleatum exerts immunosuppressive activity by inhibiting human T cell immune function, thereby promoting cancer cell proliferation (307). Furthermore, P. gingivalis can suppress host immune functions, exploit the complement system, and disrupt host immune defense responses to protect itself from attacks by humoral and cellular immune systems (308).

##### 3.5.3.2 Immune cell recruitment and polarization

Periodontal pathogens drive the polarization of tumor-associated macrophages towards the immunosuppressive M2 phenotype via chemotactic cytokines, further shaping an immune-evasive tumor microenvironment (309). Research by Tan et al. (204) demonstrated that P. gingivalis activates the CXCL1-CXCR2 axis to recruit tumor-associated neutrophils, promoting pancreatic ductal adenocarcinoma progression. Narikiyo et al. (310) found increased expression of IL-8 and GRO-α in a cell model infected with Streptococcus anginosus, activating the recruitment and activation of neutrophils and monocytes, thereby promoting esophageal cancer progression. Other researchers, through KEGG enrichment analysis, found that F. nucleatum promotes invasive behavior of cancer cells by activating the chemokine CCL20 (311).

### 3.6 Signaling pathways linking periodontal pathogens to systemic diseases

Periodontal pathogens induce systemic pathological alterations by activating host pattern recognition receptor (PRR)-mediated signaling cascades or by directly modulating intracellular pathways via virulence factors. These signals converge on immune activation, metabolic dysregulation, bone homeostasis imbalance, and cell survival programs, thereby extending localized periodontal inflammation to distant organs.

#### 3.6.1 Immune recognition and inflammation initiation

Periodontal pathogens and their components (particularly LPS) are primarily sensed by Toll-like receptors (TLRs). Porphyromonas gingivalis (P. gingivalis) LPS activates TLR2 and TLR4, which, via the MyD88-dependent pathway, activate the IKK complex, leading to IκB degradation and NF-κB nuclear translocation. This subsequently induces the transcription of key pro-inflammatory cytokines such as IL-1β, IL-6, and TNF-α (206, 309). Animal studies have shown that P. gingivalis infection exacerbates oxidative stress and systemic inflammation via the NF-κB/iNOS signaling pathway, significantly accelerating atherosclerotic plaque formation (312). A hyperglycemic environment can upregulate TLR2 expression in gingival fibroblasts, amplifying the inflammatory response, which partially explains the aggravated periodontal destruction in diabetes (313). This pathway serves as a core molecular link between periodontitis and chronic inflammatory diseases like atherosclerosis and diabetes.

#### 3.6.2 Bone homeostasis and metabolic imbalance: RANKL/OPG and AGE-RAGE axes

Periodontal bone resorption is regulated by the balance between RANKL and OPG. In patients with type 2 diabetes (especially those with HbA1c ≥8.0%), the levels of RANKL and the RANKL/OPG ratio in gingival crevicular fluid are significantly elevated, exacerbating alveolar bone loss (314, 315).

Advanced glycation end products (AGEs) and their receptor (RAGE) signaling do not directly initiate inflammation but promote the recruitment of inflammatory cells and sustained cytokine release (8). In diabetic periodontitis, AGEs activate gingival fibroblasts via the RAGE/NF-κB pathway, upregulating MMP-1 and accelerating connective tissue destruction (316). Pharmacological blockade of the AGE-RAGE signaling pathway can mitigate periodontal inflammation and bone loss in diabetic animal models (317).

#### 3.6.3 Tumor promotion and immune evasion: oncogenic and anti-apoptotic pathways

**Figure 2:**
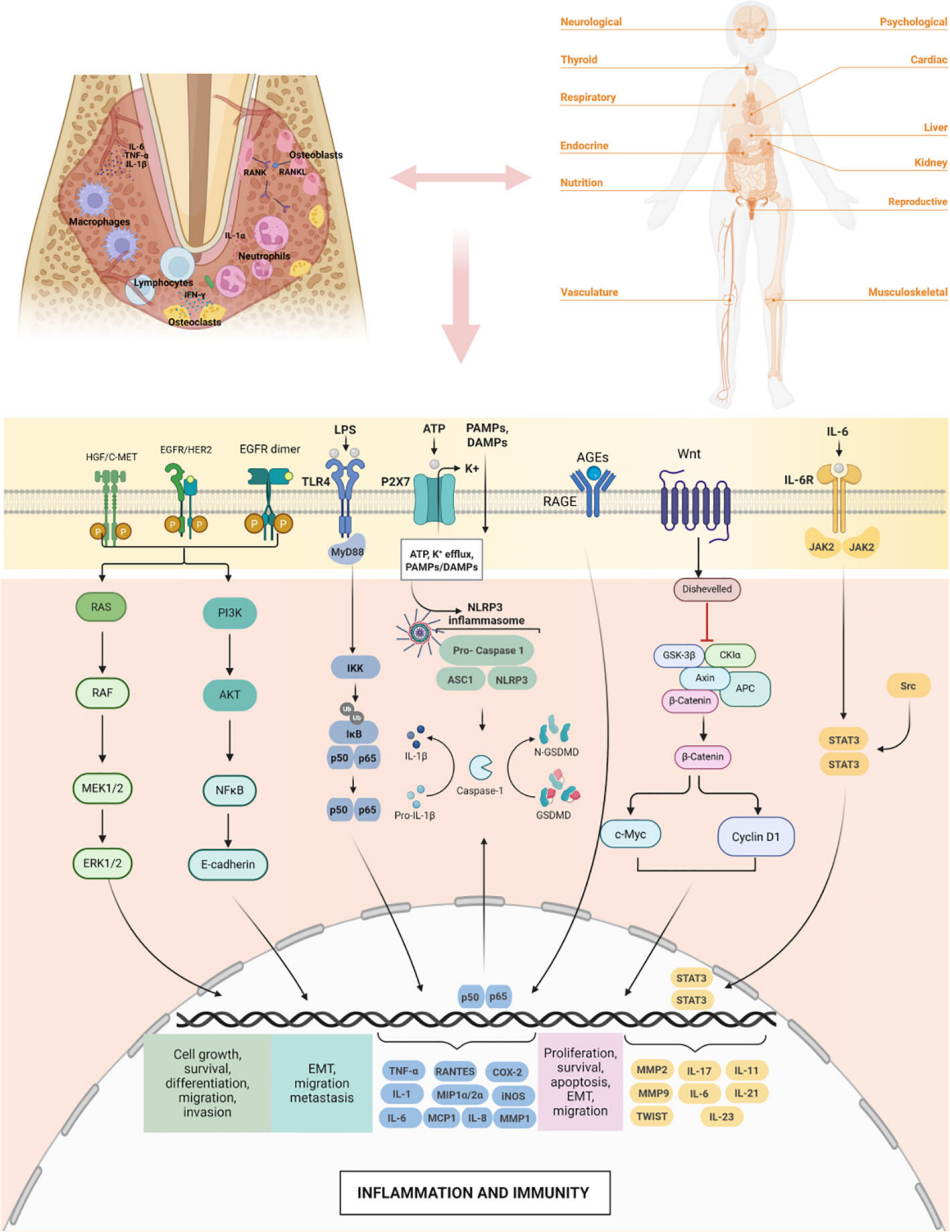
Signaling pathways linking periodontal pathogens to systemic diseases.

Periodontal pathogens promote tumorigenesis by remodeling the tumor microenvironment and directly interfering with host cell signaling pathways (**Figure 2**).

Wnt/β-catenin Signaling: The Fusobacterium nucleatum (F. nucleatum) adhesin FadA binds to epithelial E-cadherin, disrupting cell-cell junctions and activating the Wnt/β-catenin pathway. Nuclear β-catenin induces the transcription of oncogenes such as c-Myc and Cyclin D1, increasing vascular permeability and promoting tumor cell proliferation (318).

Anti-apoptotic and Proliferative Signaling: P. gingivalis enhances cell survival through multiple mechanisms: (1) Activating PI3K/Akt and inhibiting PTEN, leading to the inactivation of the pro-apoptotic protein Bad and upregulation of Bcl-2 (319); (2) Activating JAK1/STAT3, inducing the transcription of anti-apoptotic genes (320); (3) Modulating the miR-21/PDCD4/AP-1 feedback loop, resulting in Cyclin D1 overexpression and accelerated cell cycle progression (321).

Invasion and Metastasis: Gingipains activate PAR2/4, triggering ERK1/2-ETS1, p38/HSP27, and NF-κB signaling pathways. This activates pro-MMP-9 and degrades the extracellular matrix, enhancing the invasion and metastatic potential of oral squamous cell carcinoma (322, 323).

Immune Microenvironment Reprogramming: F. nucleatum Fap2 binds to the inhibitory receptor TIGIT on NK and T cells, directly suppressing cytotoxic activity (324, 325). Both P. gingivalis and F. nucleatum promote the polarization of tumor-associated macrophages towards the immunosuppressive M2 phenotype and upregulate inhibitory molecules such as PD-L1, ultimately impairing CD8^+^ T cell-mediated anti-tumor immunity (326).

#### 3.6.4 Neurological effects: neuroinflammation and cognitive impairment

Periodontal pathogens influence central nervous system homeostasis via the oral-brain axis. Experimental studies indicate that oral administration of P. gingivalis or its outer membrane vesicles (OMVs) allows entry into the brain, activating microglia. This increases pro-inflammatory cytokine secretion in human microglial clone 3 (HMC3) cells, promoting neuroinflammation and tau protein hyperphosphorylation, thereby further inducing neuroinflammation (327).

#### 3.6.5 Endocrine and vascular dysfunction

P. gingivalis OMVs can inhibit insulin-induced Akt/GSK-3β signaling in hepatocytes, reducing insulin sensitivity and interfering with insulin signaling and glucose metabolism (328). Regarding vascular function, P. gingivalis LPS promotes endothelial-mesenchymal transition through the TGF-β/Smad, ERK/MEK, PI3K, and p38 MAPK pathways, contributing to atherosclerosis (329). Its OMVs also induce vascular smooth muscle cell calcification via the ERK1/2–RUNX2 signaling pathway (330).

### 3.7 Genetics

Genome-wide association studies (GWAS) of periodontitis have demonstrated that genetic variation is one of the contributing factors to immune dysregulation associated with the disease. Significant genetic overlap has been identified between periodontitis and various bacterial diseases, cardiometabolic disorders, and autoimmune diseases, providing genetic evidence for the “oral-systemic axis.” (331) In patients with metabolic syndrome, host genetic variants, particularly single nucleotide polymorphisms (SNPs) in genes such as RUNX2, CAMTA1, and VDR—are associated with the diversity of the gingival microbiome and the abundance of specific bacteria (e.g., Porphyromonas gingivalis and Streptococcus mutans). These findings suggest that host genetic variations related to systemic metabolic health may indirectly shape the oral microbial community by modulating immune responses, a phenomenon referred to as “genetically driven dysbiosis, “ thereby further influencing the risk of periodontitis and systemic diseases (332).

### 3.8 Epigenetics

#### 3.8.1 Atherosclerosis and cardiovascular disease

The core mechanism by which periodontitis accelerates atherosclerosis lies in its induction of pro-inflammatory epigenetic reprogramming in monocytes/macrophages. Studies reveal that stimulation with Porphyromonas gingivalis (P. gingivalis) lipopolysaccharide (LPS) or live bacteria can induce lasting alterations in histone modifications at the promoter regions of inflammation-related genes (e.g., TNFA, IL6) in monocytes. These changes include, for example, an increase in H3K4me3 (an activating mark) and a decrease in H3K9me3 (a repressive mark). This modified state can be maintained even after the stimulus is removed, rendering the cells hyperresponsive to subsequent pro-atherogenic stimuli (e.g., ox-LDL), leading to increased production of pro-inflammatory cytokines and accelerating plaque development (333). Animal models confirm that periodontal infection can enhance histone acetylation levels within macrophages of the aortic root plaques in atherosclerosis-prone mice (334).

#### 3.8.2 Type 2 diabetes and insulin resistance

Chronic inflammation is a key driver of insulin resistance, with histone modifications acting as downstream effectors of inflammatory signaling pathways such as NF-κB. Under the combined influence of hyperglycemia and chronic inflammatory factors (e.g., TNF-α), pathways like NF-κB in insulin target tissues (adipose, liver) and immune cells become persistently activated. Activated NF-κB recruits histone acetyltransferases (e.g., p300) to the promoter regions of genes involved in promoting inflammation and gluconeogenesis (e.g., Pck1), resulting in sustained histone acetylation and gene overexpression, forming a “metabolic memory” (335). When periodontitis is concurrent, the persistent systemic inflammatory burden it generates can further exacerbate systemic insulin resistance and metabolic dysregulation. This process is believed to involve an aggravation of the aforementioned epigenetic programming (31).

#### 3.8.3 Rheumatoid arthritis

Periodontitis and RA share an autoimmune inflammatory background, with histone citrullination representing a unique intersection point. The periodontal pathogen P. gingivalis is not only the sole human pathogen known to express PPAD (peptidylarginine deiminase), but its infection can also upregulate the activity of the host’s own PAD enzymes (e.g., in neutrophils). PADs catalyze the citrullination of arginine residues on histones H3 and H4. Citrullinated histones not only alter their own function but can also serve as novel autoantigens recognized by autoantibodies, thereby breaking immune tolerance and contributing to RA pathogenesis (336). This provides direct molecular evidence supporting the concept that “periodontal infection is an environmental trigger for RA.”

#### 3.8.4 Tumor initiation and progression

Systemic inflammation associated with periodontitis may influence the tumor microenvironment via histone modifications. Beyond the classic mechanisms of direct bacterial dissemination and inflammatory mediator spread affecting distal tumorigenesis, epigenetic reprogramming, particularly alterations in histone modifications, is garnering increasing attention. Chronic inflammation itself is a key driver of aberrant epigenetic programming associated with tumors (337). Periodontitis possesses a unique capacity for epigenetic intervention; its major pathogen, P. gingivalis, secretes proteases capable of directly cleaving histones, and microbial metabolites such as butyrate are known histone deacetylase (HDAC) inhibitors (248). Such local interventions may have systemic consequences. Recent research indicates that periodontitis can induce long-term, pro-inflammatory epigenetic reprogramming in bone marrow hematopoietic stem cells (a phenomenon termed ‘trained immunity’), leading to the persistent production of functionally altered myeloid cells (334). These cells, upon entering the circulation, may infiltrate distant tumor sites and, through their abnormal histone modification status and gene expression profiles, contribute to the formation of an immunosuppressive tumor microenvironment. Furthermore, drawing from gut microbiome research, the paradigm where microbial metabolites influence host immunity and cancer by modulating histone modifications has been established (338), suggesting that periodontal dysbiosis may participate in systemic epigenetic regulation via similar pathways.

#### 3.8.5 Post-transcriptional regulation by non-coding RNAs

Non-coding RNAs (ncRNAs), particularly microRNAs (miRNAs), play a central role in post-transcriptional gene regulation and serve as crucial mediators linking periodontal inflammation to systemic diseases. Within the inflammatory microenvironment of periodontitis, the circulating and exosomal miRNA profiles undergo significant alterations. These miRNAs can be transported via extracellular vesicles to distant organs, where they modulate insulin sensitivity, metabolic homeostasis, and vascular function, thereby establishing a molecular bridge between periodontitis and the pathologies associated with obesity, diabetes, and hypertension.

Distinct miRNA expression patterns have been observed in individuals with obesity and concomitant periodontitis. Perri et al. reported that in obese periodontitis patients, miR-150, miR-24, and miR-27a were upregulated, while members of the let-7 family and miR-26a-5p were downregulated, suggesting synergistic dysregulation in pathways related to adipogenesis, insulin signaling, and immune activation (339). Notably, there is increased expression of virally derived miRNAs (particularly those encoded by human herpesviruses) in the gingival tissues of obese periodontitis patients. For instance, HHV-8-derived miR-K12-4-3p may indirectly influence periodontal inflammation and metabolic dysfunction by competing with host miRNAs for shared targets or by regulating viral latency and replication (340).

Beyond metabolic diseases, shared miRNA signatures have also been identified between periodontitis and cardiovascular conditions. Rodriguez et al. (341) identified 13 miRNAs associated with both periodontitis and hypertension, including miR-100-5p, miR-21-5p, miR-34a-5p, miR-146a-5p, miR-26b-5p, miR-126-3p, miR-181a-5p, miR-15b-5p, miR-223-5p, miR-16-5p, miR-30a-5p, and miR-17-5p. These miRNAs are predominantly known regulators of endothelial function, vascular inflammation, and smooth muscle cell behavior, highlighting the convergence of post-transcriptional regulatory networks in the periodontal-cardiovascular interplay (**Table 2**).

**Table 2:** ncRNA-mediated post-transcriptional regulation linking periodontitis to systemic diseases.

ncRNA/miRNA	Targeted pathways or molecules	Associated systemic diseases	Major mechanisms of action	References
miR-146a	IRAK1, TRAF6/NF-κB	Diabetes mellitus, systemic inflammation	Dysregulation of negative feedback control of NF-κB signaling, resulting in imbalanced inflammatory responses and amplification of systemic inflammation	Perri R et al, 2012 (339)
miR-150	Inflammatory and immune regulatory pathways	Obesity with periodontitis	Associated with immune cell differentiation and metabolic inflammation; upregulation reflects amplification of chronic inflammatory responses	Naqvi A R et al, 2019 (340)
miR-24	MAPK/inflammatory signaling	Obesity, metabolic disorders	Regulates inflammation- and lipid metabolism–related pathways, contributing to metabolic–inflammatory crosstalk	Naqvi A R et al, 2019 (340)
miR-27a	Adipogenesis and insulin signaling	Obesity, insulin resistance	Modulates adipocyte differentiation and energy metabolism, thereby exacerbating metabolic imbalance	Naqvi A R et al, 2019 (340)
let-7 family	Insulin signaling, cell cycle regulation	Obesity, diabetes mellitus	Downregulation leads to reduced insulin sensitivity and enhanced chronic inflammation	Naqvi A R et al, 2019 (340)
miR-26a-5p	Inflammatory and metabolic homeostasis pathways	Obesity, metabolic syndrome	Downregulation is associated with disruption of metabolic homeostasis and persistence of inflammatory responses	Naqvi A R et al, 2019 (340)
miR-K12-4-3p	Host inflammation-related genes	Obesity with periodontitis	Competitively targets host genes with cellular miRNAs, regulating viral latency and indirectly modulating inflammation and metabolism	Perri R et al, 2012 (339)
miR-21-5p	NF-κB, PTEN	Hypertension, cardiovascular diseases	Regulates vascular inflammation and endothelial function, contributing to the development of hypertension	Rodriguez N M ea al, 2024 (341)
miR-100-5p	mTOR signaling/vascular homeostasis	Hypertension	Affects vascular smooth muscle cell function and inflammatory responses	Rodriguez N M ea al, 2024 (341)
miR-34a-5p	Cellular senescence and inflammatory pathways	Hypertension, cardiovascular diseases	Promotes endothelial dysfunction and a pro-inflammatory senescent phenotype	Rodriguez N M ea al, 2024 (341)
miR-126-3p	Endothelial repair and angiogenesis	Hypertension	A key endothelial-protective miRNA; downregulation indicates impaired vascular homeostasis	Rodriguez N M ea al, 2024 (341)
miR-223-5p	Immune cell activation	Hypertension, systemic inflammation	Regulates inflammatory responses of granulocytes and macrophages	Rodriguez N M ea al, 2024 (341)
miR-15b-5p/miR-16-5p	Cell cycle and inflammatory regulation	Hypertension	Influences vascular cell proliferation and inflammatory signaling	Rodriguez N M ea al, 2024 (341)
miR-17-5p/miR-30a-5p	TGF-β/inflammatory pathways	Hypertension	Participates in vascular remodeling and maintenance of chronic inflammation	Rodriguez N M ea al, 2024 (341)
miR-181a-5p	Immune regulation and metabolic signaling	Hypertension	Modulates immune activation thresholds and affects vascular inflammation	Rodriguez N M ea al, 2024 (341)
miR-26b-5p	Metabolic and inflammatory pathways	Hypertension	Associated with regulation of metabolic homeostasis and vascular function	Rodriguez N M ea al, 2024 (341)

## 4 Treatment

**Figure 3:**
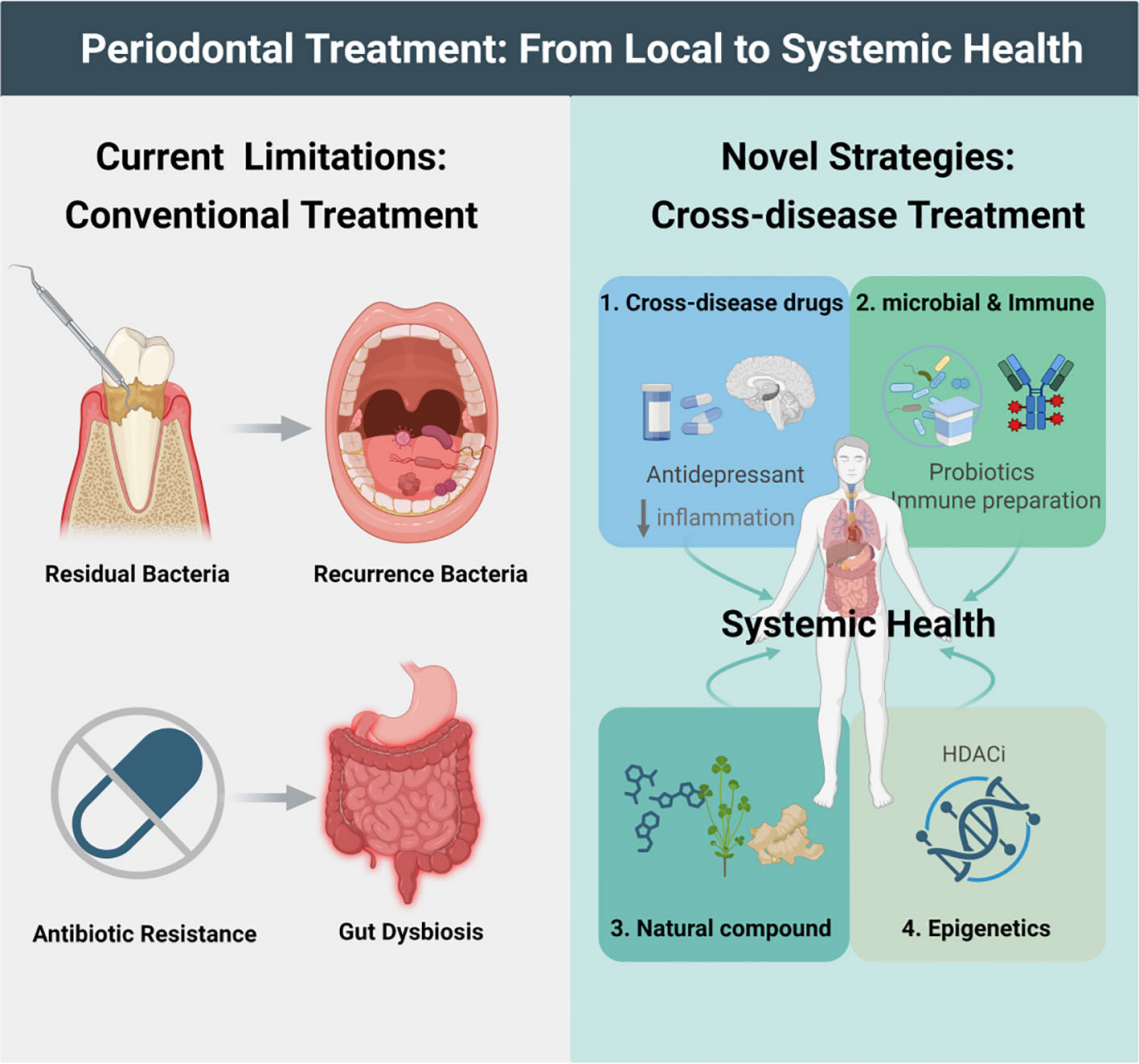
Periodontal treatment: from local to systemic health.

Current research indicates that intensive periodontal therapy can improve both periodontal and systemic conditions by removing subgingival plaque. However, despite interventions such as scaling and root planing procedures, adjunctive antibiotic regimens, and supportive periodontal therapy (SPT), periodontitis remains prone to recurrence. Moreover, long-term use of antibiotics can lead to side effects such as gastrointestinal issues and drug resistance (342–344). Therefore, a holistic approach exploring anti-inflammatory drugs with cross-disease applicability, microbial-targeted and immunomodulatory therapies, and natural compounds may offer more possibilities for treating the comorbidity of periodontitis and systemic diseases (**Figure 3**).

### 4.1 Cross-disease therapeutic application of anti-inflammatory drugs

Inflammation is a common pathological basis for periodontitis and many systemic diseases. Drugs with anti-inflammatory properties, particularly antidepressants, have been explored as adjunctive treatments for periodontitis.

For example, the tricyclic antidepressant (TCA) tianeptine, in a rat model of depression (induced by olfactory bulbectomy) combined with periodontitis, not only improved depressive behavior but also significantly reduced periodontal tissue destruction and alveolar bone resorption. Its mechanisms involve stimulating hippocampal neurogenesis, lowering lipopolysaccharide (LPS)-induced pro-inflammatory cytokines (e.g., TNF-α), and increasing the production of anti-inflammatory factors (e.g., TGF-β1 and IL-10), thereby modulating the host’s immune response to bacterial challenge (345).

Another TCA, desipramine, has been shown to reduce alveolar bone loss in rats with experimental periodontitis. Its protective effects are associated with downregulating key inflammatory mediators (IL-1β, inducible nitric oxide synthase, cyclooxygenase-2) in periodontal tissues, as well as inhibiting dendritic cell antigen presentation and T-lymphocyte proliferation—cells that are important regulators of periodontal bone remodeling (346).

Furthermore, the selective serotonin reuptake inhibitor (SSRI) fluoxetine has demonstrated both anti-inflammatory and antidepressant effects. A clinical cross-sectional study found that depression patients receiving fluoxetine treatment had better periodontal clinical parameters (bleeding on probing, clinical attachment loss) compared to non-medicated patients. Animal experiments further indicate that fluoxetine alleviates periodontal inflammation and bone destruction by reducing IL-1β expression in gingival tissues and inhibiting NF-κB transcriptional activity and matrix metalloproteinase-9 (MMP-9) production (347).

### 4.2 Microbial-targeted and immunomodulatory therapies

Targeted therapies against key periodontal pathogens and the abnormal immune responses they trigger are a current research focus. For instance, developing monoclonal antibodies against virulence factors of specific pathogens has shown efficacy in inhibiting periodontal destruction in experimental periodontitis models (348). Given the central role of dysbiosis, using probiotics, prebiotics, or specific antimicrobial peptides to restore the ecological balance of the oral microbiota is a promising “ecological therapeutic” approach (349).

Moreover, the timely resolution of inflammation is as crucial as its initiation. Research reveals that endogenous pro-resolving mediators (e.g., lipoxins) can not only actively terminate inflammation and clear tissue debris but also enhance mucosal defense, preventing the entry of periodontal pathogens into the bloodstream. This points to a direction for developing novel drugs that mimic or enhance these endogenous pathways (350).

### 4.3 Therapeutic potential of natural compounds

Natural compounds have garnered significant attention for their multi-target and low-toxicity profile in regulating periodontitis and related systemic diseases.

Curcumin: As one of the most widely studied natural anti-inflammatory agents, curcumin effectively inhibits the cytotoxicity of Porphyromonas gingivalis outer membrane vesicles (OMVs). It dose-dependently reduces OMV-induced secretion of inflammatory cytokines (IL-6, IL-1β, TNF-α) from epithelial cells and inhibits OMV adhesion to and invasion of host cells, thereby protecting tissue integrity (351).

Tanshinone IIA: Studies have found that in an atherosclerosis model with P. gingivalis infection, Tanshinone IIA reduces reactive oxygen species (ROS) generation by downregulating NADPH oxidase (NOX2/4) and inhibits the NF-κB signaling pathway. This ultimately lowers systemic and vascular oxidative stress and inflammation, slowing the progression of atherosclerosis (352).

Anethole: Research by Yoshino N et al. shows that anethole, abundant in fennel, exerts a dual antimicrobial effect by depleting essential nutrient transport proteins (RagA/RagB) of P. gingivalis and inhibiting its protease activity (353).

### 4.4 Epigenetic regulation: a new perspective with HDAC inhibitors

Recent research has highlighted the critical role of epigenetic modifications in chronic inflammatory diseases. Aberrant hyperactivity of histone deacetylases (HDACs) is associated with persistent inflammation and bone destruction. Therefore, HDAC inhibitors are being explored as an emerging therapeutic strategy.

Cantley MD et al. demonstrated that topical application of broad-spectrum or selective HDAC inhibitors (e.g., butyrate, trichostatin A) in experimental periodontitis models exhibits anti-inflammatory and bone-protective effects. The mechanisms involve restoring antimicrobial peptide expression and promoting M2 macrophage polarization. Notably, HDAC inhibitors (e.g., vorinostat) are already clinical drugs for certain hematological malignancies and have shown efficacy in models of autoimmune diseases and atherosclerosis (354).

Treatment with MS-275 significantly reduced local accumulation of immune cells and mRNA levels of representative pro-inflammatory molecules in prostate tissue. It increased the percentage of Foxp3^+^ CD4^+^ Treg cells in lymph nodes and their proportion among peripheral blood CD4^+^ cells. *In vitro* studies indicated that MS-275 could induce a relative increase in ED2^+^ macrophages and shift macrophage polarization from the classical M1 phenotype to the anti-inflammatory M2 phenotype (355).

Furthermore, butyrate, an HDAC inhibitor produced by gut microbiota through dietary fiber fermentation, has been shown to alleviate systemic inflammation and improve metabolic health (248).

## 5 Conclusion and future perspective

Mounting evidence supports the concept of the oral cavity as a critical hub and sentinel for systemic health, rather than an isolated anatomical site. Within the framework of the “oral-X axis, “ this review systematically integrates epidemiological, microbiological, immunological, and mechanistic studies to demonstrate the interplay between oral chronic inflammation and systemic diseases.

Mechanistically, periodontitis represents a source of sustained microbial and inflammatory burden. Initiated by pathogen-dominated oral dysbiosis, it triggers a cascade of events including inflammatory responses, epithelial barrier disruption, endotoxemia, oxidative stress, and immune reprogramming. Beyond direct bacteremia, oral microbes disseminate to distant organs via ectopic colonization through routes such as the gastrointestinal tract, respiratory tract, placental circulation, and immune cell-mediated “Trojan horse” mechanisms. Critically, persistent low-grade inflammation and trained immunity may imprint long-term epigenetic and functional changes in innate immune cells. This provides a mechanistic basis for the chronic, self-perpetuating nature of oral-systemic disease interactions, even after local microbial control.

From a systemic perspective, the oral-X axis should be viewed as a dynamic, bidirectional network where oral inflammation not only exacerbates systemic disease susceptibility but is itself shaped by systemic metabolic, immune, and hormonal disturbances. This mutual reinforcement may partly explain the frequent comorbidity of periodontitis with multiple conditions and the often-limited efficacy of single-organ therapeutic approaches. Reconceptualizing periodontitis as a systemic inflammatory amplifier rather than a localized infection reshapes its role in precision medicine and chronic disease management.

Despite substantial progress, several critical gaps remain. Most current evidence is correlative, and causal inference requires well-designed prospective studies, interventional trials, and integrated multi-omics analyses. Furthermore, microbial heterogeneity across populations, disease stages, and host genetic backgrounds complicates the identification of universal pathogenic signatures. Standardization of periodontal phenotyping and the integration of functional microbial and host immune readouts are essential for advancing the field.

From a translational viewpoint, the oral-X axis offers an evidence base for early intervention and risk stratification. A clinical study found that periodontitis is independently associated with concentric left ventricular remodeling, suggesting that cardiovascular evaluation should be considered for patients with chronic periodontitis, particularly those with concomitant metabolic syndrome (356). Periodontal status and oral microbial signatures may serve as convenient biomarkers for systemic disease risk assessment. Moreover, growing clinical evidence suggests that intensive periodontal therapy and targeted modulation of the oral-gut-immune axis can attenuate systemic inflammation and improve outcomes in specific systemic diseases. Future therapeutic strategies may move beyond mechanical debridement to include host modulators, microbiome-based interventions, and natural bioactive compounds aimed at restoring immune homeostasis rather than merely eradicating pathogens.

In summary, redefining oral health as an integral component of systemic disease prevention and management carries profound implications for both clinical practice and public health. Interdisciplinary collaboration among dentistry, internal medicine, immunology, and systems biology will be crucial for translating mechanistic insights from the oral-X axis into personalized and preventive healthcare strategies.
